# Blood Pressure Management for Acute Intracerebral Hemorrhage: A Meta-Analysis

**DOI:** 10.1038/s41598-017-13111-x

**Published:** 2017-10-30

**Authors:** Ligen Shi, Shenbin Xu, Jingwei Zheng, Jing Xu, Jianmin Zhang

**Affiliations:** 1 Department of Neurosurgery, Second Affiliated Hospital, School of Medicine, Zhejiang University, Hangzhou, Zhejiang, China; 2 Brain Research Institute, Zhejiang University, Hangzhou, Zhejiang, China; 3 Collaborative Innovation Center for Brain Science, Zhejiang University, Hangzhou, Zhejiang, China

## Abstract

Inconsistent data from the randomized trials ignites controversy on intensive blood pressure (BP) reduction for acute intracerebral hemorrhage (ICH). This study aims to examine the association between BP lowering and clinical outcomes among patients with acute ICH. We conducted this meta-analysis based on the published randomized controlled trials (RCTs). Data were included from 6 RCTs involving 4412 patients. No significant improvements were observed in hematoma growth at 24 hours, neurologic improvement at 24 hours, hypotension at 72 hours, death or dependency at 90 days, mortality at 90 days, and serious adverse events at 90 days between intensive and conservative BP lowering groups. High heterogeneity was observed between estimates in hematoma growth (*I* ^2^ = 49). Univariate meta-regression and subgroup analysis showed that intensive BP lowering showed a significant decrease in hematoma growth in age ≤62 years, time from symptoms onset to treatment ≤6 hours, baseline hematoma volume ≤15 mL, and combined intraventricular hemorrhage ≤25% subgroups. In conclusion, intensive BP management in patients with ICH is safe. Intensive BP lowering could reduce hematoma growth in those patients (≤62 years old) with ICH volume less than 15 mL receiving BP management within 6 hours after randomization.

## Introduction

Spontaneous intracerebral hemorrhage (ICH) affects 2.5 per 10,000 people worldwide annually^1^, and is associated with a high mortality that only 38 percent of ICH patients could survive over one year^2^. Early blood pressure (BP) elevation occurs in more than 90% of affected patients^3^. Extremely elevated BP is reported to predict hematoma expansion and poor neurological functional outcomes^4^. Observational data showed a beneficial effect of early intensive BP lowering in patients with ICH^5,6^. However, it has been reported that excessively low admission systolic BP (SBP) might cause cerebral hypoperfusion and ultimately lead to poor outcomes^7^. Whether rapid BP lowering in patients with acute ICH would reduce hematoma expansion and improve final outcomes remains on debate.

Current AHA/ASA (American Heart Association/American Stroke Association) guidelines recommended that acute lowering of SBP to 140 mm Hg is safe for those ICH patients with SBP between 150 and 220 mm Hg (*Class I; Level of Evidence A*)^8^. And it can be effective for improving functional outcome (*Class IIa; Level of Evidence B*)^8^. For those ICH patients with SBP >220 mm Hg, aggressive BP reduction should be managed using a continuous intravenous infusion with frequent BP monitoring (*Class IIb; Level of Evidence C*). These recommendations were based primarily on the data from the phase Intensive Blood Pressure Reduction in Acute Cerebral Hemorrhage II (INTERACT-2) trial enrolling 2839 ICH patients presenting with SBP between 150 and 220 mm Hg within 6 hours^9^. This trial observed that intensive BP lowering had no effect on reducing the primary outcome of death or major disability, but it could enhance physical functioning compared with conservative BP lowering treatment^9^. However, this trial was argued for its various use of available antihypertensive drug with different mechanisms, in which the effects might have varied across different agents^10^. For example, calcium channel blockers could relieve vasospasm to improve cerebral perfusion. In the INTERACT-2 trial, approximately 16.2% of ICH patients received a calcium channel blocker in the intensive BP lowering group compared with 8.5% of ICH patients in the conservative treatment group^10^. The Antihypertensive Treatment of Acute Cerebral Hemorrhage II (ATACH-2) trial was designed to provide additional information on the efficacy of intravenous nicardipine for intensive BP lowering in patients within 4.5 hours after symptom onset^11^. However, this trial was discontinued for futility before achieving the target enrollment of 1280 ICH patients^11^. Moreover, a high occurrence of serious adverse events at 90 days was observed in the intensive BP lowering group in this ATACH-2 trial^11^.

In consideration of these inconsistent data from the former trials, we aimed to conduct a meta-analysis to examine the association between BP lowering and clinical outcomes among patients with acute ICH.

## Methods

This meta-analysis followed the PRISMA (Preferred Reporting Items for Systematic Reviews and Meta-Analyses) format guidelines^12^.

### Search Strategy and Information Sources

All RCTs reporting the efficacy and safety of intensive BP lowering in patients with acute ICH were enrolled from three major databases, MEDLINE, EMBASE, and the Cochrane Library, by two independent investigators (LS and SX). The following search strategy was used in MEDLINE: ((intracranial hemorrhage [Title/Abstract]) OR (intracerebral hemorrhage [Title/Abstract])) AND (blood pressure [Title/Abstract]). Similar search strategy was performed for EMBASE and the Cochrane Library databases from January 2000 to November 2016 without language or other restrictions. In addition, Reference lists of all RCTs, reviews, comments, and meta-analysis were examined to ensure that no relevant studies had been missed by the database search.

### Study Selection and Data Collection

Only studies with acute ICH patients who randomly assigned to receive intensive or conservative BP reduction treatment were included in this meta-analysis. Two independent investigators (LS and SX) scanned all studies to select applicable studies. Case reports or series, retrospective or prospective observational studies, and RCTs without control groups were excluded from the final analysis. Data on eligibility criteria, study design, baseline characteristics of the participants, and outcome assessments from the included trials were extracted independently by two investigators (LS and SX).

### Outcomes Definition and Quality Assessment

Short-term outcomes were assessed with hematoma growth and neurologic improvement at 24 hours, and hypotension at 72 hours. Hematoma growth was defined as the proportion of acute ICH patients with ≥33% hematoma expansion on the computed tomography (CT) scan at 24 hours compared with the admission scan. Neurologic improvement was defined as an increase of ≥2 points in the Glasgow Coma Score (GCS) or a decrease of ≥4 points in the National Institutes of Health Stroke Scale (NIHSS), which was sustained for at least 8 hours within 24 hours after randomization. Hypotension was defined as the proportion of participants who required therapy with intravenous vasopressor drugs within 72 hours after randomization.

Long-term outcomes were included death or dependency, mortality, and serious adverse events at 90 days. Modified Rankin scale (mRS) runs from 0 to 6 scores in consistent with perfect health without symptoms to death^13^. Dependency was defined as a score of 3 to 5 on the mRS scale at 90 days after randomization. Serious adverse events were included renal failure, recurrent stroke, acute coronary event, severe hypotension, and other life-threatening events.

Biases of the included trials were assessed by 2 independent investigators (LS and SX) using a 7-point quality control recommended by Cochrane Handbook^14^. The items contained selection bias, performance bias, detection bias, attrition bias, reporting bias, and other potential biases. Each items was categorized as high, low, or unclear risks.

### Data Synthesis and Analysis

All data were calculated by STATA (*Version 1*2.*0*). Odds ratios (ORs) and 95% credibility interval (CI) were calculated to express the safety and effect of intensive BP reduction in patients with acute ICH compared with conservative BP lowering treatment. A random-effects model and z test were used to calculate the pooled ORs. A *P* value of less than 0.05 was considered statistically significant. Heterogeneity was assessed with the Cochran Q and *I* ^2^ statistics. High heterogeneity was defined as *I* ^2^ values of ≥50%. Univariate meta-regression (Method of Moments) and subgroup analysis were performed to evaluate sources of heterogeneity. Publication bias was assessed using Egger’s funnel plot with pseudo 95% confidence limits.

## Results

### Study Selection and Characteristics

**Figure 1:**
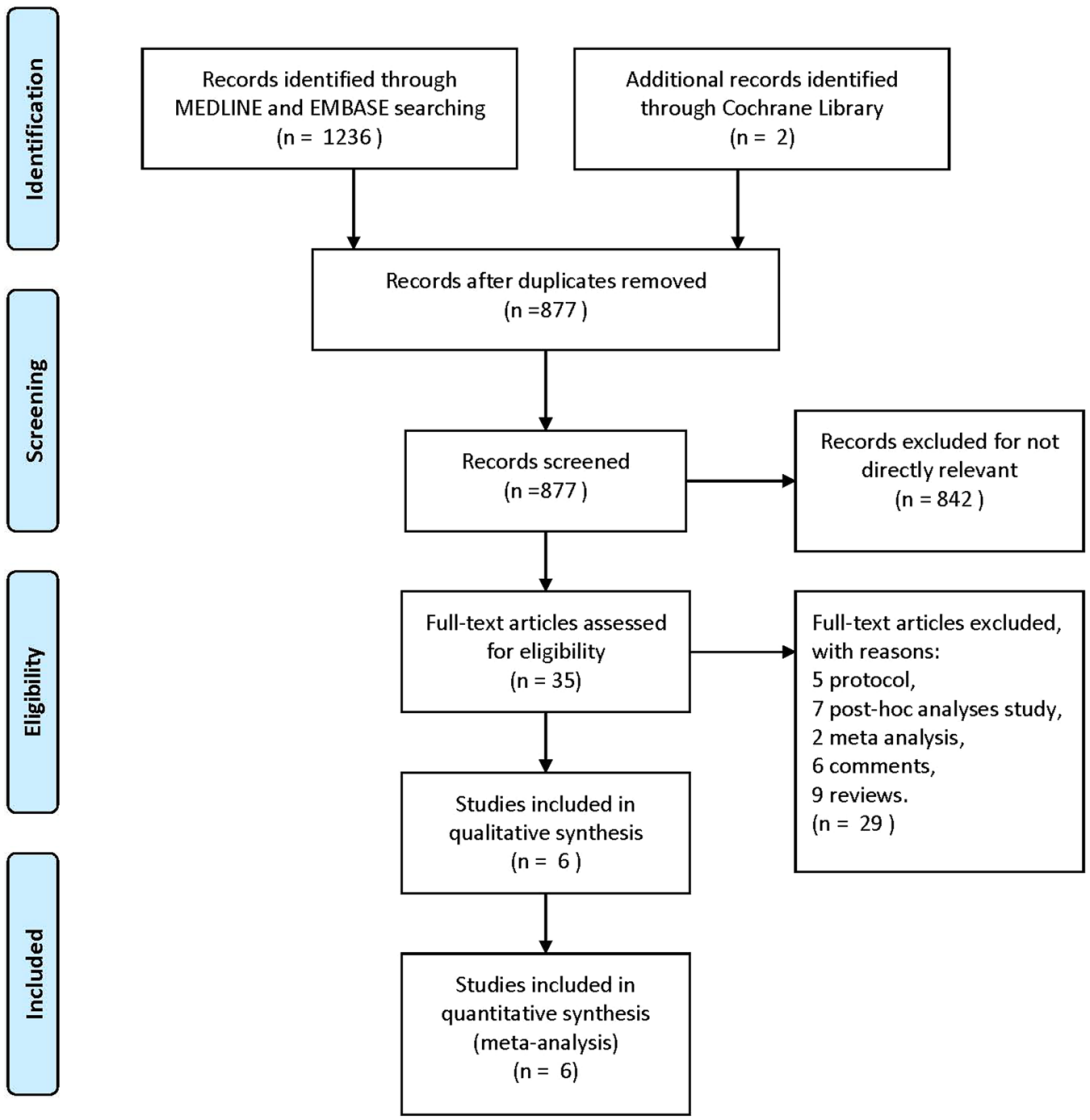
The study search, selection, and inclusion process.

MEDLINE, EMBASE and Cochrane Library databases were searched for all records reporting the efficacy and safety of intensive BP lowering in patients with acute ICH, from which we obtained 35 records without duplicates. Protocols, post-hoc analyses studies, meta-analysis, comments, and reviews were excluded after assessing full-text articles. Ultimately, six studies (ATACH-2 2016^11^, GONG 2015^15^, INTERACT-2 2013^9^, ADAPT 2013^16^, INTERACT 2008^17^, and KOCH 2008^18^) were included in quantitative synthesis (Fig. 1). The characteristics of the included trials are summarized in Table 1.

**Table 1:** Descriptive Summary of Included Randomized Trials Characteristics.

Cohort	Time Frame for Treatment	Baseline Blood Pressure	Antihypertensive Drugs	Intensive Blood-Pressure Lowering				Conservative Blood-Pressure Lowering				Follow-up times
				**No. of Patients**	**Baseline NIHSS Score**	**Baseline Hematoma Volume**	**Target blood pressure**	**No. of Patients**	**Baseline NIHSS Score**	**Baseline Hematoma Volume**	**Target blood pressure**	
ATACH-2, 2016	4.5 hours	SBP: 180–240 mmHg	Immediately intravenous nicardipine and maintain for 24 hours	500	11 (0–40)	10.3 (2.3–85.2)	SBP: 110–139 mmHg	500	11 (0–40)	10.2 (0.98–79.1)	SBP:140–179 mmHg	90 days
GONG, 2015	4 hours	SBP ≥ 160 mmHg	Various intravenous antihypertensive drugs for 24 hours	60	9.74 (4.49)	10.86 (5.72)	SBP: 110–139 mmHg	60	9.50 (4.81)	11.02 (5.67)	SBP:140–179 mmHg	14 days
INTERACT-2, 2013	6 hours	SBP: 150–220 mmHg	Various intravenous antihypertensive drugs within 1 hour and maintain for 7 days	1399	10 (6–15)	15.7 (15.7)	SBP: 110–139 mmHg	1430	11 (6–16)	15.1 (14.9)	SBP:140–179 mmHg	90 days
ADAPT, 2013	24 hours	SBP ≥ 150 mmHg	Immediately intravenous labetalol and maintain for 24 hours	39	10 (6–18)	25.98 (30.84)	SBP: 110–149 mmHg	36	11 (5.5–15.5)	26.86 (25.24)	SBP:150–179 mmHg	90 days
INTERACT, 2008	6 hours	SBP: 150–220 mmHg	Various antihypertensive drugs were administrated within 1 hour and maintain for 7 days	174	9 (5–14)	14.2 (14.5)	SBP: 110–139 mmHg	172	9 (5–16)	12.7 (11.6)	SBP:140–179 mmHg	90 days
KOCH, 2008	8 hours	MAP ≥ 110 mmHg	Intravenous labetalol or nicardipine for 48 hours	21	12 (7.0)	12.5 (17.2)	MAP:110–130 mmHg	21	10.9 (6.5)	8.5 (9.8)	MAP < 110 mmHg	90 days

NIHSS: National Institutes of Health Stroke Scale; SBP: Systolic Blood Pressure; MAP: Mean Arterial Blood Pressure.

### Overall and subgroup analysis

**Figure 2:**
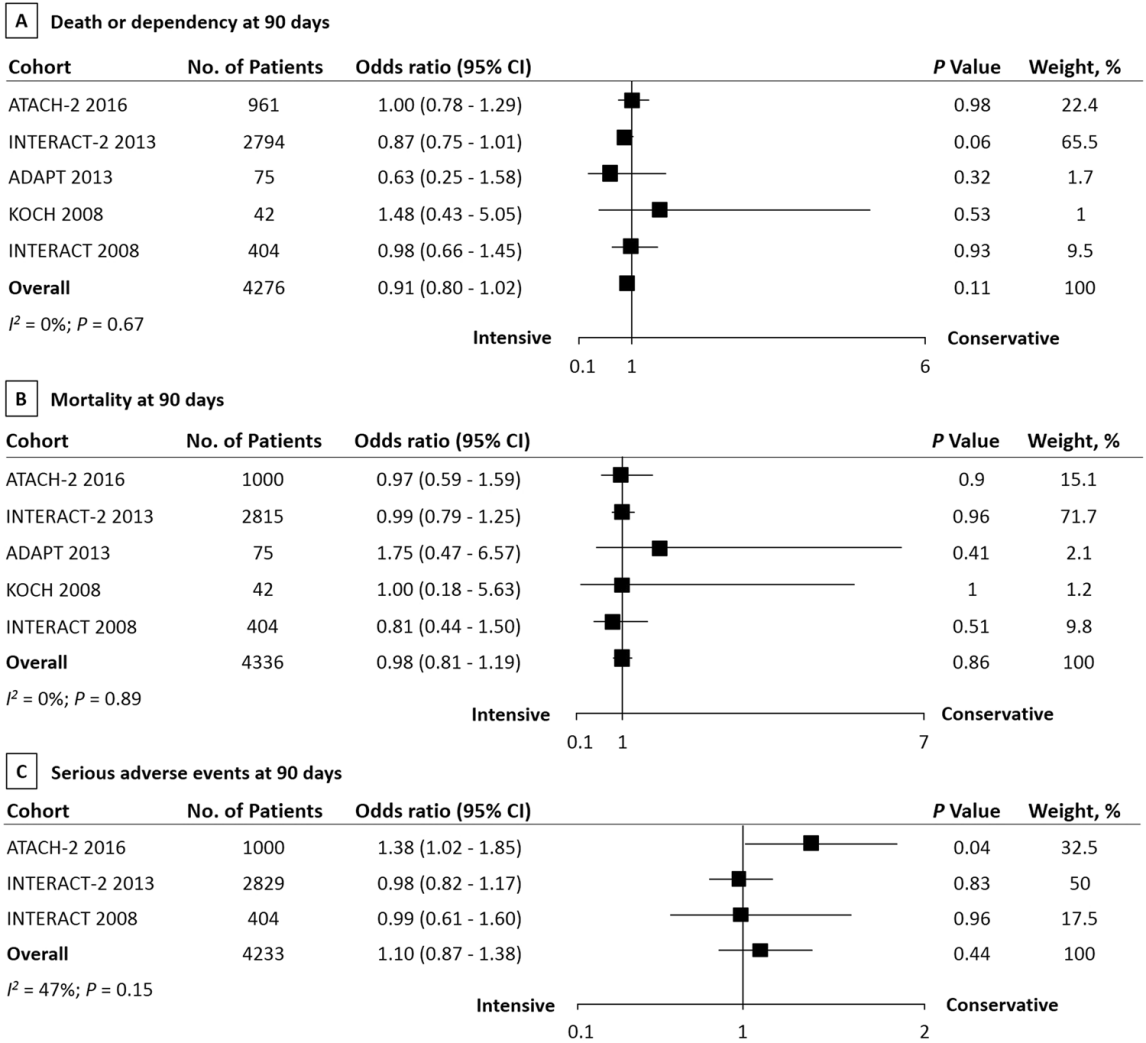
The pooled relative risk of the short-term outcomes. The diamond indicates the estimated relative risk (95% confidence interval) for all patients.

For long-term outcomes, intensive BP reduction showed no significant differences in death or dependency at 90 days (OR 0.91, 95% CI 0.80 to 1.02, *P = *0.11; Fig. 2A), mortality at 90 days (OR 0.98, 95% CI 0.81 to 1.19, *P = *0.86; Fig. 2B), and serious adverse events at 90 days (OR 1.10, 95% CI 0.87 to 1.38, *P = *0.44; Fig. 2C) compared with conservative BP lowering treatment in patients with acute ICH. No evidence of heterogeneities were observed between estimates in death or dependency at 90 days (*I* ^2^ = 0%; *P* = 0.67) and mortality at 90 days (*I* ^2^ = 0%; *P* = 0.89). But a moderate heterogeneity was observed in serious adverse events at 90 days (*I* ^2^ = 47%; *P* = 0.15).

**Figure 3:**
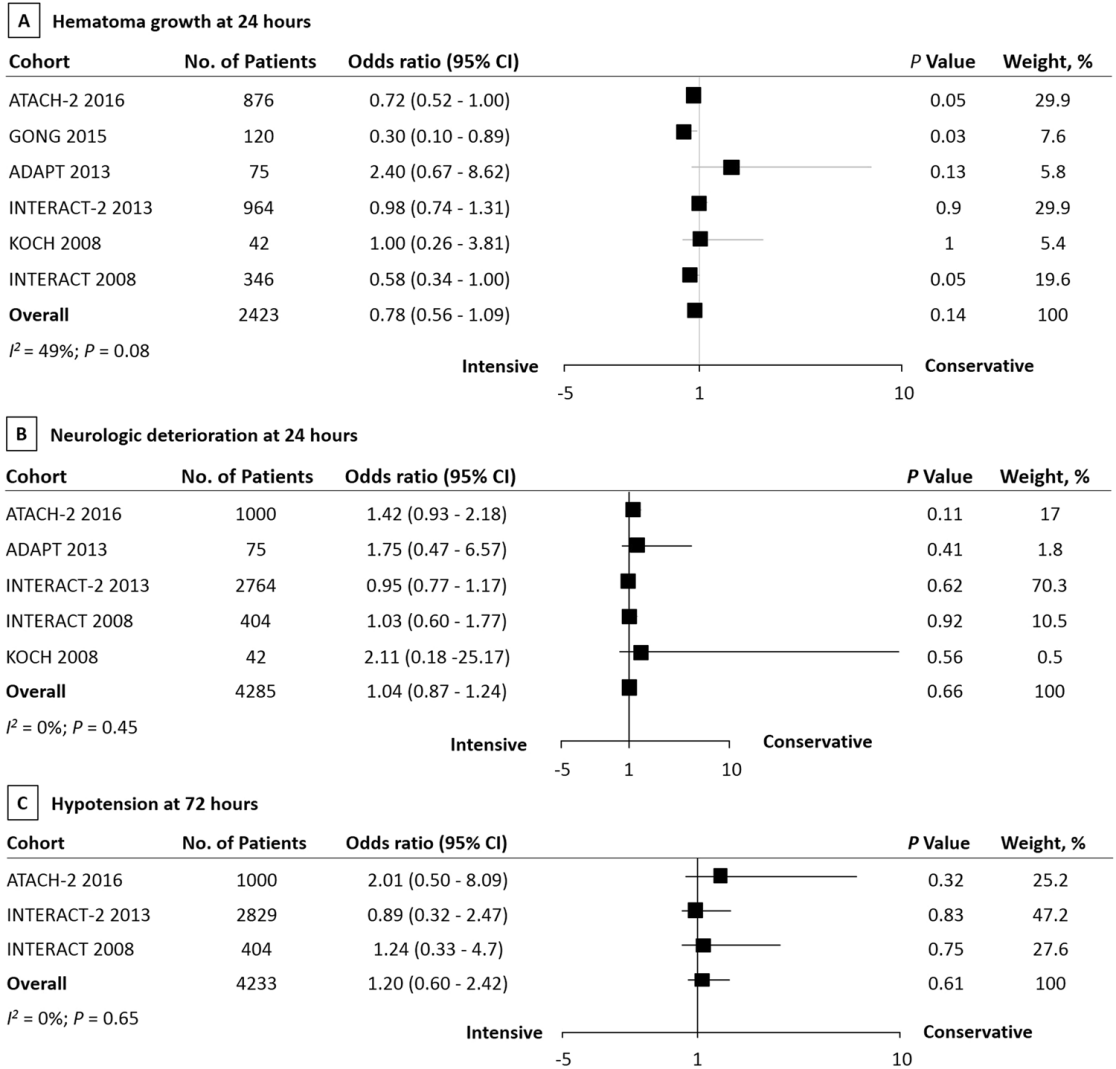
The pooled relative risk of the long-term outcomes. The diamond indicates the estimated relative risk (95% confidence interval) for all patients.

For short-term outcomes, no significant differences were observed between intensive and conservative BP lowering groups in hematoma growth at 24 hours (OR 0.78, 95% CI 0.56 to 1.09, *P = *0.14; Fig. 3A), neurologic deterioration at 24 hours (OR 1.04, 95% CI 0.87 to 1.24, *P = *0.66; Fig. 3B), and hypotension at 72 hours (OR 1.20, 95% CI 0.60 to 2.42, *P = *0.61; Fig. 3C). No evidence of heterogeneities were observed between estimates in neurologic deterioration at 24 hours (*I* ^2^ = 0%; *P* = 0.45) and hypotension at 72 hours (*I* ^2^ = 0%; *P* = 0.65). But a moderate heterogeneity was observed in hematoma growth at 24 hours (*I* ^*2*^ = 49%; *P* = 0.08).

**Figure 4:**
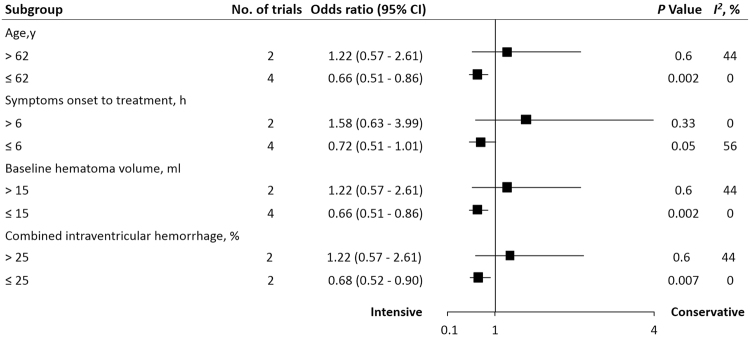
The pooled relative risk of the outcomes in subgroup analysis. The diamond indicates the estimated relative risk (95% confidence interval) for all patients.

Univariate meta-regression showed no independent predictor (*P* > 0.05) of hematoma growth (Table 2). In subsequent subgroup analysis, intensive BP reduction was associated with a great reduction of hematoma growth in age ≤62 years (OR 0.66, 95% CI 0.51 to 0.86, *P = *0.002), time from symptoms onset to treatment ≤6 hours (OR 0.72, 95% CI 0.51 to 1.01, *P = *0.05), baseline hematoma volume ≤ 15 mL (OR 0.66, 95% CI 0.51 to 0.86, *P = *0.002), and combined intraventricular hemorrhage (IVH) ≤ 25% (OR 0.68, 95% CI 0.52 to 0.90, *P = *0.007) subgroups (Fig. 4).

**Table 2:** Univariate meta-regression analyses evaluating the association of baseline characteristics with 24-hour hematoma enlargement.

Factors	Point estimate	95% CI	*P* Value
Symptoms onset to treatment	1.071	0.969, 1.184	0.129
Symptoms onset to target blood pressure	1.056	0.970, 1.149	0.149
Rapid lowing blood pressure	1.021	0.905, 1.151	0.630
Hypertension	0.024	2.41e-13, 2.38e + 09	0.591
Baseline NIHSS score	1.352	0.798, 2.289	0.187
Age	1.190	0.998, 1.418	0.051
Baseline hemotoma volume	1.085	0.966, 1.219	0.122
Baseline blood pressure	0.984	0.917, 1.056	0.555
Combined intraventricular hemorrhage	7390	0.0009, 6.39e + 10	0.138

NIHSS: National Institutes of Health Stroke Scale.

### Risk of bias for independent studies

**Figure 5:**
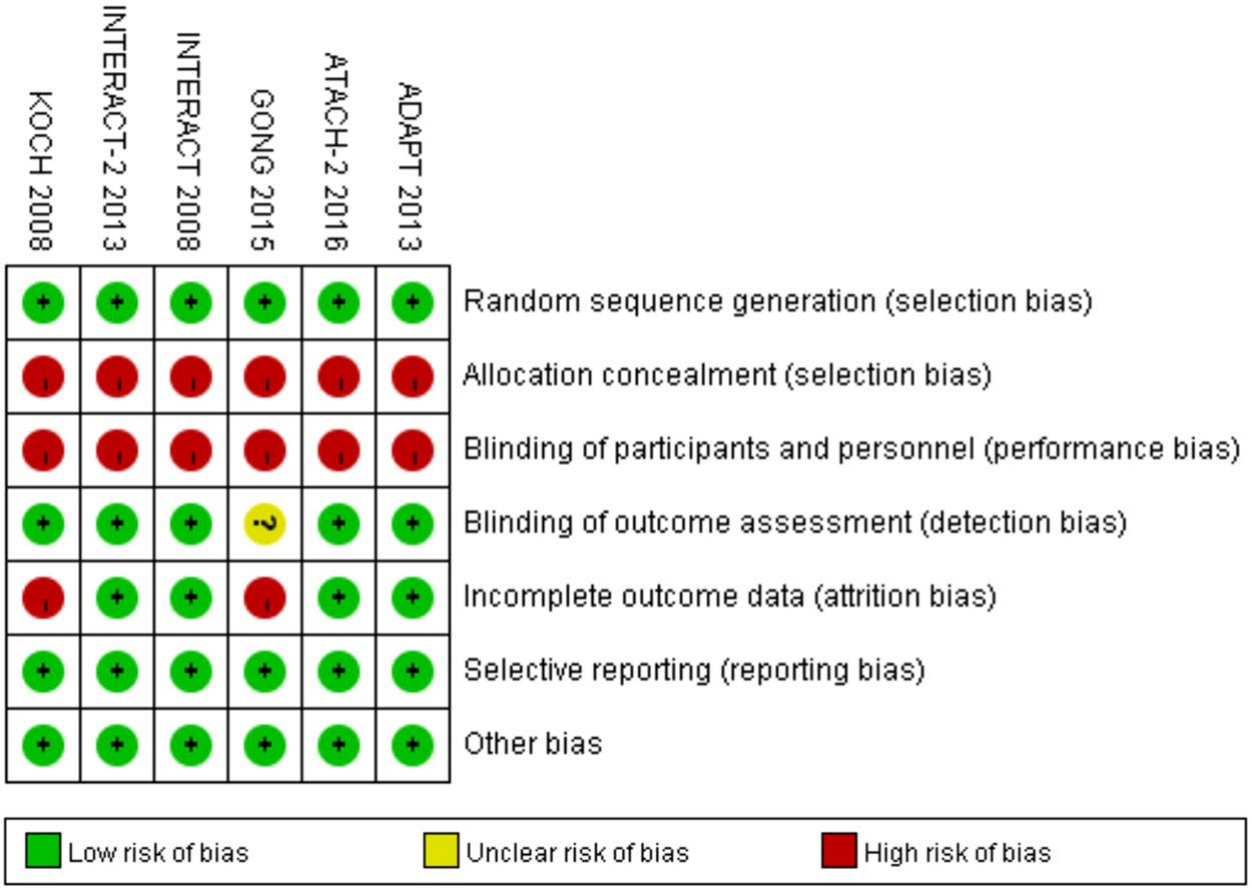
Risk of bias: A summary table for each risk of bias item for each study.

Risk of bias in the included studies is summarized in Fig. 5. All the included trials were open-label RCTs. Except for the GONG 2015 trial^15^, all trials stated that they were blind to assess outcomes. The KOCH 2008^18^ and GONG 2015^15^ trials were lack of data on adverse events at 90 days after randomization. Publication bias was detected using Egger’s funnel plot with pseudo 95% confidence limits, which showed low risks (data not shown).

## Discussion

The data from the present meta-analysis showing similar incidence of 72-hour hypotension and 3-month serious adverse events between intensive and conservative BP reduction groups, indicated that intensive BP lowering treatment was safe (target SBP <140 mm Hg or Mean Arterial Blood Pressure [MAP] <110 mm Hg) in patients with acute ICH. Intensive BP lowering therapy did not appear to have curative effects on either 24-hour neurologic improvement or 3-month functional outcome (death or dependency). But it might have a considerable attenuation of hematoma growth in those patients (≤62 years old) with ICH volume less than 15 mL receiving BP management within 6 hours.

The first issue is the safety of intensive BP lowering in patients with acute ICH. The evidence from the present meta-analysis is reassuring. However, the heterogeneity of 3-month serious adverse events was 47% with *P* value of 0.15. Univariate meta regression or subgroup analysis was not applicable due to the limited included trials. The ATACH-2 trial observed a higher occurrence of 7-day renal adverse events and 3-month serious adverse events in those patients assigned to the intensive lowering group than those assigned to the conservative BP lowering group^11^. Renal adverse events might be associated with hypoperfusion, although the occurrence of 72-hour hypotension showed no significant difference between intensive and conservative BP lowering groups^11^. In view of these former data, intensive BP lowering treatment is acceptable for those patients with acute ICH.

The second question is the efficacy of lowering BP in improving the functional outcomes. The raise in BP levels is very common after acute ICH onset. Several potential mechanisms have been involved in this pathological process, including increase intracranial pressure, premorbid hypertension, neuro-endocrine, and activation of neuro-vegetative signaling pathways^19^. High BP levels in acute ICH patients have been associated with intracranial pressure elevation, cerebral edema formation, and hematoma expansion^20,21^. Hematoma expansion in the early phase of ICH strongly predicted poor long-term outcomes^22^. Intensive BP lowering was regarded as an effective management for controlling hematoma expansion^5,6^. However, this used to be a concern whether rapid BP lowering in patients with acute ICH would cause global or regional cerebral hypoperfusion, especially in the perihematoma. The ADAPT trial found that intensive BP lowering has no significant impact on perihematoma cerebral blood flow^16^, which was also consistent with previous observational studies^23^. The results of the present meta-analysis indicated that intensive BP reduction has no significant effect on either 24-hour hematoma growth or 3-month functional outcome. Subgroup analysis indicated that age, therapeutic time window, baseline hematoma volume, and combined with IVH were associated with hematoma expansion at 24 hours after randomization. Among these factors, age was reported as an independent predictor of neurologic recovery^24^. In the univariate meta-regression analysis, only age showed a potential association with hematoma expansion (*P* = 0.05). Larger volume of baseline hematoma combined with IVH showed worse outcomes. Analysis of previous available data indicated that each 1 mL growth in hematoma might increase a 7% risk of death or disability^25^. Spontaneous ICH combined IVH showed 51% risk of death compared to 20% without IVH^26^. Our findings supported the hypothesis that there is a time-dependent loss of benefit in the intensive BP lowering treatment. ICH might have a wider therapeutic window than acute ischemic stroke, due to its lack of ischemic penumbra^27^. The ADAPT trial^16^ indicated a similar effect of intensive BP lowering on hematoma expansion between ≤3 hours and ≤ 4.5 hours. Our data showed that intensive BP lowering could reduce hematoma growth within 6 hours after randomization. In addition, previous meta-analysis including four trials^9,16–18^ indicated that baseline NIHSS score was an independent predictor of 3-month unfavorable outcome (death or dependency)^28^. However, in the present univariate meta-regression analysis, baseline NIHSS score was not associated with hematoma expansion.

Several limitations of this meta-analysis need to be acknowledged. The first issue is high selection and performance biases that all the included trials were open-label RCTs. Although five of all the included trials were outcome-blinded, performance biases still cannot be ruled out. The limited trials including in this meta-analysis made it impossible to perform multivariate meta regression analysis to detect the interaction among these influential factors. Variable antihypertensive medications with different mechanisms were used in the included trials. In addition, the large size of the INTERACT-2 trial results in disproportional weights in effect sizes.

In conclusion, the present meta-analysis indicated that intensive BP management in patients with ICH is safe, but has no contribution to 90-day neurological functional recovery. Intensive BP lowering could reduce hematoma growth in those patients (≤62 years old) with ICH volume less than 15 mL receiving BP management within 6 hours after randomization.
